# Supplementary material for: A novel expert-annotated single-cell dataset for thyroid cancer diagnosis with deep learning benchmarks
Source: PLOS Digit Health. 2025 Dec 16;4(12):e0001120. doi: 10.1371/journal.pdig.0001120 (PMC12707618; doi:10.1371/journal.pdig.0001120)
Supplement: Attachment — Submitted filename: Response to Plos Reviewers.docx [file pdig.0001120.s001.docx]

**Response to Reviewers**

Manuscript Title: A Novel Expert-Annotated Single-Cell Dataset for Thyroid Cancer Diagnosis with Deep Learning Benchmarks

Manuscript ID: PDIG-D-25-00315

Journal Name: PLOS Digital Health

Dear Dr. Assi, Prof. Celi, and Reviewers,

We sincerely thank you for your time and effort in reviewing our manuscript titled “*A Novel Expert-Annotated Single-Cell Dataset for Thyroid Cancer Diagnosis with Deep Learning Benchmarks*” submitted to PLOS Digital Health. We are deeply grateful for the insightful and constructive comments provided, which have greatly helped us improve the quality and clarity of our work.

We would also like to note that, after further revision with our clinical collaborators, we confirmed that releasing the dataset does not conflict with institutional or ethical regulations. All cytological images were fully anonymized and cropped to exclude any patient-identifiable information. Accordingly, we have made the dataset publicly available on Zenodo(DOI:10.5281/zenodo.17226934), along with the full codebase and documentation on Github(<https://github.com/nqHuynq/hybrid-cell-classification>). This update revises our previous statement regarding controlled access and ensures full compliance with PLOS’ data policy.

In this revised version, we have carefully addressed all reviewer suggestions. Changes to the manuscript are highlighted in blue to distinguish the revised content from the original text. We hope that these revisions meet your expectations and further strengthen the contribution of our study.

We look forward to hearing from you regarding our submission and remain available to respond to any further questions or clarifications.

Sincerely,
Thanh-Ha Do
On behalf of all authors
12/09/2025

**Reviewer 1**

**Comment 1:**

*Clearly confirm that the dataset (and ideally model code) is publicly accessible via a repository, with documentation.*
**Response:**

Thank you for highlighting the importance of accessibility and reproducibility. The dataset originates from a clinical study conducted at the 108 Military Central Hospital and it has now been publicly released on Zenodo with a permanent DOI:10.5281/zenodo.17226934 . The repository includes the full collection of 3,419 single-cell images annotated with nine nuclear morphological features, along with accompanying documentation.

In parallel, we have made all possible technical resources openly available to support reproducibility. Specifically, we have released the whole codebase, including baseline implementations and the proposed pipeline, along with pretrained model weights on GitHub. For convenience, the pretrained models have also been uploaded to Hugging Face, enabling straightforward use in future studies. Comprehensive documentation is provided to facilitate replication and extension of our work.

**Comment 2:** *Include inter-observer agreement or annotation consistency metrics if possible, to validate annotation quality.*
**Response:**

We recognize that annotation quality is central to the reliability of our study. Annotation was carried out by a team of seven cytopathologists with 5–20 years of experience, led by Dr. De, Head of the Department of Pathology at the 108 Military Central Hospital and the author of this study, who has over 20 years of expertise. To protect patient confidentiality, we used an in-house annotation tool that only retained anonymized single-cell images, with all other metadata masked. The nine nuclear features were defined based on expert consensus and Bethesda guidelines. Two cytopathologists independently annotated each image in a cross-over design; in cases of disagreement, a third expert adjudicated, and the final label was decided by group consensus.

To address the reviewer’s suggestion, we have expanded **Section 3.2 (Page 6-7)** to describe this annotation protocol in detail. Although formal inter-observer agreement metrics were not computed due to resource constraints, the structured cross-validation and adjudication process, which was directly supervised by the corresponding author, provides a high degree of annotation consistency. We believe this clarification strengthens the transparency of our dataset construction and reassures readers about label reliability.

**Comment 3:** *Add a brief discussion on the dataset’s generalizability and its limitations due to the single-institution source.*
**Response:**

We agree that the generalizability of the dataset is an important consideration. The data were collected over more than two years within a large referral center, covering patients of different ages, genders, and clinical conditions. The high case volume and diversity of the sampled population enhance the representativeness of the dataset at the national level and ensure that a wide spectrum of cytopathological features is captured.

At the same time, we acknowledge the inherent limitations of sourcing data from a single institution. Laboratory-specific factors such as staining protocols, slide preparation, and imaging equipment may differ across sites and introduce domain shifts when models are applied elsewhere. Moreover, the dataset primarily reflects characteristics of the Vietnamese population, which may limit its transferability to other ethnic or regional groups. Nonetheless, the models and framework we provide constitute a solid foundation for transfer learning, and can be adapted to additional datasets when such resources become available.

**Comment 4:** *Consider extending the ethical discussion to address future clinical applications and implications.*
**Response:**

We appreciate this critical comment. Ethical considerations are central to the responsible use of Deep Learning in healthcare. The dataset used in this study was approved by the Institutional Review Board of the 108 Military Central Hospital, and the protocols for patient privacy and data anonymization were strictly followed. Only de-identified single-cell images were retained, with all personal and clinical metadata removed.

The primary goal of our work is to develop decision-support tools that assist cytopathologists in cancer diagnosis, rather than replacing human experts. Such systems could also serve as valuable educational resources for training junior pathologists. Any future deployment in clinical settings would require approval under the national regulations governing the use of AI in healthcare, ensuring compliance with both ethical and legal standards.

We envision that models like ours could be integrated into telepathology platforms to support remote diagnostics, particularly in under-resourced regions. This could expand access to high-quality diagnostic expertise while maintaining strict oversight and accountability. We have revised the manuscript to discuss these future implications in **Section 6 (Page 23-24)**.

**Reviewer 2**

**Comment 1:** *Limited Discussion of Model Limitations: While the study highlights high false positive rates for certain features due to visual ambiguities (section 4.4), it does not sufficiently discuss specific limitations of the ConvNeXt model or potential reasons for misclassifications beyond visual similarity (e.g., dataset size constraints or feature extraction challenges).*

*Recommendation: Expand the discussion in Section 4.5 to include a deeper analysis of model limitations, such as the impact of the dataset’s relatively small size (3,419 images) or potential overfitting to common labels. Suggest specific strategies for future improvements (e.g., transfer learning or larger datasets).*

**Response:**

We thank the reviewer for this constructive comment. We agree that the limitations of our model extend beyond visual ambiguity and require a deeper analysis. In particular, the relatively small dataset size (3,419 single-cell images) inevitably restricts the ability of the model to capture the full spectrum of morphological variability, and the persistent class imbalance biases predictions toward common features while reducing sensitivity to rare but clinically meaningful labels. In addition, certain nuclear patterns such as grooves and irregular contours are inherently challenging to separate due to overlapping visual characteristics, and this difficulty is further exacerbated by the relatively low resolution of cropped single-cell images (80×80 pixels), which can obscure fine structural details. Even though ConvNeXt is effective at extracting global texture and shape, its feature representations may not fully disentangle these subtle variations. These factors collectively help explain the observed misclassifications and highlight that errors cannot be solely attributed to visual ambiguity. To reflect this analysis, we have expanded **Section 4.4 (Page 18)** to provide a more comprehensive discussion of these dataset-related and architectural constraints.

We also recognize the importance of explicitly situating these issues within the broader limitations of our study. Beyond dataset size and class imbalance, the single-institution source of our data introduces potential domain shift when applying models trained under different staining protocols, slide preparation methods, or imaging devices. Furthermore, while Grad-CAM visualizations provide helpful intuition, they remain qualitative tools that cannot guarantee definitive attribution of model decisions. Together, these constraints underscore the need for future work on enlarging and diversifying the dataset, applying morphology-preserving and synthetic augmentation to strengthen rare labels, and leveraging transfer learning or multi-institutional collaborations to improve generalizability. To clarify these points and better align with the reviewer’s recommendation, we have revised **Section 5 (Page 23)** to expand the limitations discussion and to outline these directions for future improvement explicitly.

**Comment 2:** *Future Work Specificity: The future work section mentions improving calibration for rare labels and expanding the dataset but lacks specificity on how these will be achieved.*
**Response:**

We thank the reviewer for raising this important point. We agree that the original description of future work was too general, and we have now provided more concrete directions to strengthen this section.

First, for rare-label calibration, we will systematically evaluate post-hoc methods such as temperature scaling and isotonic regression, applied directly to the sigmoid outputs of our multi-label classifier. These methods will be benchmarked with calibration metrics including expected calibration error (ECE), reliability diagrams, and rare-label F1-score. This aims to reduce overconfidence and improve the trustworthiness of probability estimates in clinical settings.

Second, to overcome the current dataset limitations, we plan multi-institutional collaborations to collect new samples obtained under diverse staining protocols, slide preparation procedures, and imaging devices. Standardized metadata and strict quality control will be applied before integration, thereby expanding the dataset size and reducing domain bias. With this richer dataset, we will also revisit attention-based models such as Swin Transformers, which currently underperform due to limited training data but may show competitive results once sufficient samples are available.

Third, we will explore advanced augmentation strategies to address rare-class underrepresentation. In particular, we intend to implement morphology-preserving techniques such as elastic deformations and stain normalization, as well as generative approaches like GAN-based synthetic sample creation for severely rare morphologies (e.g., intranuclear inclusions). Cytopathologists will validate all synthetic images to ensure biological plausibility before inclusion in training.

Finally, ensemble and fusion methods represent a promising future direction. Since different backbones capture complementary morphological cues, we will investigate weighted ensemble averaging and trainable late fusion at the logit level.

These methods will be systematically validated using cross-validation, with a focus on rare-label sensitivity and calibration quality, aiming to enhance both robustness and generalizability. These additions provide a structured and detailed roadmap for future research. We have incorporated this expanded discussion in **Section 6 (Pages 23–24)**.

**Comment 3:** *The use of Grad-CAM visualizations is a highlight, as it bridges the gap between technical performance and clinical interpretability, which should be emphasized in the revised manuscript.*
**Response:**

We thank the reviewer for highlighting this important point. We fully agree that Grad-CAM visualizations are not only a useful interpretability tool but also play a critical role in bridging technical performance with clinical relevance. In our revised manuscript, we emphasize that Grad-CAM provides an additional layer of transparency by revealing whether the model’s focus aligns with cytologically meaningful regions, such as nuclear structures, rather than irrelevant background artifacts. This alignment reassures both readers and clinicians that the model is learning biologically valid features instead of spurious correlations introduced during preprocessing. For example, we observed that Grad-CAM consistently highlights nuclei while ignoring the neutral background, indirectly validating our single-cell cropping strategy. Such interpretability is especially crucial in medical applications, where model trustworthiness directly impacts clinical acceptance.

We also note that Grad-CAM visualizations help reduce the “black-box” perception of deep learning models by allowing clinicians to cross-check the model’s reasoning against established diagnostic criteria. Although qualitative in nature, these attention maps provide a valuable bridge between computational predictions and human expertise, thereby supporting safer translation of computational pathology tools into practice. To better reflect this perspective, we have expanded our discussion of Grad-CAM in **Section 4.5 (Page 22)**, highlighting its role in fostering clinical interpretability and trust.

**Comment 4:** *Include a limitation section.*
**Response:**

We thank the reviewer for this valuable suggestion. We also note that this point partly overlaps with Comment 1, as both highlight the importance of carefully acknowledging the study’s limitations. To avoid redundancy, we have structured these discussions in a more systematic way within **Section 5: Limitations** of the revised manuscript.

To begin with, we address dataset-related constraints. The dataset, though carefully annotated, is relatively small (3,419 images) and originates from a single institution. This may limit generalizability to other hospitals where staining protocols or imaging devices differ, raising the possibility of domain shift.

In addition, we discuss the issue of class imbalance. Despite implementing several mitigation strategies, performance for rare but clinically significant features remains lower than for common ones, reflecting persistent challenges in handling underrepresented classes.

We further recognize limitations arising from visual ambiguity and image quality. Cropped single-cell images are of relatively low resolution, which can obscure subtle morphological details, while overlapping nuclear features may contribute to misclassification.

Lastly, we highlight that Grad-CAM, while useful for interpretability, is inherently qualitative. Its outputs provide plausibility checks rather than definitive evidence of feature attribution, and should be interpreted with caution.

These points are now explicitly discussed in **Section 5: Limitations (Page 23)** of the revised manuscript.

## **Additional Clarifications**

In addition to addressing all reviewer comments, we have corrected minor typographical and grammatical errors throughout the manuscript.
All changes in the revised manuscript are highlighted in blue for clarity.
